# Estrogen receptor β represses Akt signaling in breast cancer cells via downregulation of HER2/HER3 and upregulation of PTEN: implications for tamoxifen sensitivity

**DOI:** 10.1186/bcr2865

**Published:** 2011-04-14

**Authors:** Karolina Lindberg, Luisa A Helguero, Yoko Omoto, Jan-Åke Gustafsson, Lars-Arne Haldosén

**Affiliations:** 1 Department of Biosciences and Nutrition, Novum, Karolinska Institutet, Blickagången 6, S-141 83 Huddinge, Sweden; 2 Department of Organic Chemistry and Natural Products, University of Aveiro, Avenida da Universidade, 3810-193 Aveiro, Portugal; 3 Center for Nuclear Receptors and Cell Signaling, Department of Biology and Biochemistry, 3605 Cullen Boulevard, SERC Bldg 545, University of Houston, Houston, TX 77204-5056, USA

## Abstract

**Introduction:**

The inhibition of estrogen receptor (ER) α action with the ER antagonist tamoxifen is an established treatment in the majority of breast cancers. *De novo *or acquired resistance to this therapy is common. Expression of ERβ in breast tumors has been implicated as an indicator of tamoxifen sensitivity. The mechanisms behind this observation remain largely uncharacterized. In the present study, we investigated whether ERβ can modulate pathways implicated in endocrine resistance development.

**Methods:**

T47-D and MCF-7 ERα-expressing breast cancer cells with tetracycline-regulated expression of ERβ were used as a model system. Expression levels and activity of known regulators of endocrine resistance were analyzed by performing quantitative polymerase chain reaction assays, Western blot analysis and immunostaining, and sensitivity to tamoxifen was investigated by using a cell proliferation kit.

**Results:**

Expression of ERβ in ERα-positive T47-D and MCF-7 human breast cancer cells resulted in a decrease in Akt signaling. The active form of an upstream regulator of Akt, proto-oncogene *c-ErbB-2*/receptor tyrosine kinase erbB-3 (HER2/HER3) receptor dimer, was also downregulated by ERβ. Furthermore, ERβ increased expression of the important inhibitor of Akt, phosphatase and tensin homologue deleted on chromosome 10 (PTEN). Importantly, ERβ expression increased the sensitivity of these breast cancer cells to tamoxifen.

**Conclusions:**

Our results suggest a link between expression of ERβ and endocrine sensitivity by increasing PTEN levels and decreasing HER2/HER3 signaling, thereby reducing Akt signaling with subsequent effects on proliferation, survival and tamoxifen sensitivity of breast cancer cells. This study supports initiatives to further investigate whether ERβ presence in breast cancer samples is an indicator for endocrine response. Current therapies in ERα-positive breast cancers aim to impair ERα activity with antagonists or by removal of endogenous estrogens with aromatase inhibitors. Data from this study could be taken as indicative for also using ERβ as a target in selected groups of breast cancer.

## Introduction

Approximately two-thirds of breast cancers express estrogen receptors (ERs) and initially require estrogen to grow, and are therefore treated with ER antagonists, such as tamoxifen, or by depletion of endogenous estrogens with aromatase inhibitors [1,2]. Two ERs, ERα and ERβ, have been identified [3]. ERα plays an important role in the proliferation and progression of breast cancer, whereas a distinct function of ERβ in breast cancer initiation and development has not yet been clearly established. In *in vitro *settings, ERβ inhibits proliferation, migration and invasion of breast cancers cells [4-9] as well as the growth of breast tumor xenografts [10].

ERα is the marker of choice to decide endocrine treatment of breast cancer. However, in the case of tamoxifen treatment, despite the initial response to the therapy, one-third of patients will acquire resistance even though their ERα status may remain unchanged [11]. ERβ has also been considered a marker of endocrine response. Lower expression of ERβ is found in tamoxifen-resistant tumors, and high levels of ERβ are sometimes associated with a better clinical outcome in ERα-expressing breast tumors [12]. However, some studies have indicated that in high-grade, ERα-negative, node-positive breast tumors, ERβ presence appears to be a marker related to a more aggressive breast cancer [13].

Breast tumors overexpressing receptor tyrosine kinases (RTKs) are less likely to benefit from tamoxifen treatment [14-17]. Receptor tyrosine protein kinase erbB-3 (HER3) and proto-oncogene *c-ErbB-2 *(HER2) are members of the epidermal growth factor receptor (EGFR) family. HER3 lacks intrinsic kinase activity and relies on heterodimerization with other members of the EGFR family for transduction of signals. There is growing awareness of the importance of HER2/HER3 heterodimer formation in breast cancer progression, where coexpression of HER2 and HER3 has been shown to be a poor prognostic indicator associated with resistance to endocrine therapy and to HER tyrosine kinase inhibitors [18-22]. The majority of HER2-positive tumors are strongly positive for HER3 [18], which is also seen in mouse models of breast cancers, where high expression of HER2 is commonly associated with activated and overexpressed HER3 [23]. Furthermore, inhibition of HER2 correlates with reduction in HER3 phosphorylation [24] and, correspondingly, inhibition of HER3 reduces phosphorylation of HER2 and abrogates HER2-mediated tamoxifen resistance [25].

Phosphatidylinositol 3-kinase (PI3K) promotes generation of phosphatidylinositol (3,4,5)-triphosphate (PIP3), which leads to phosphorylation and activation of the serine/threonine kinase Akt. The PI3K/Akt pathway plays important roles in regulating cell proliferation, growth, apoptosis and motility. Increased activity due to genetic changes is frequently seen in breast cancer, resulting in tumor progression, metastases and resistance to endocrine treatment [26-29]. Mutation of the *PIK3CA *gene, which encodes the p110α catalytic subunit of PI3K, leads to activation of Akt and is found in 18% to 40% of human breast cancers [30-32]. Stimulation of RTKs also activates Akt [33-35], and overexpression of HER2 is linked to elevated Akt activities [36-38]. In ERα-positive breast cancers treated with tamoxifen, detection of activated Akt at diagnosis has been shown to correlate to decreased overall survival [39].

Constitutive active Akt is also associated with loss of phosphatase and tensin homologue deleted on chromosome 10 (PTEN) expression [40]. PTEN is a tumor suppressor whose expression is often lost in breast cancers and associated with poor disease outcome [41-44]. PTEN antagonizes PI3K activity by dephosphorylating PIP3, resulting in lower levels of active Akt [45].

The goal of this study was to investigate whether ERβ1 (referred to hereinafter as ERβ) has any effect on the RTK/PI3K/Akt signaling pathway and thereby represents a regulator of tamoxifen sensitivity. We show that in ERα-positive breast cancer cells, expression of ERβ reduced Akt activation through downregulation of HER2/HER3 signaling and upregulation of PTEN and, importantly, increased sensitivity to tamoxifen. ERβ has sometimes been suggested as a predictor of endocrine response; however, the mechanisms underlying this response are still unknown. Here we suggest a link between expression of ERβ and endocrine sensitivity.

## Materials and methods

### Cell cultures

T47-D cells with tetracycline-regulated expression of ERβ485 (T47-DERβ) [4] were routinely grown not expressing ERβ (-ERβ) in RPMI 1640 medium (Invitrogen, Paisley, United Kingdom) supplemented with 5% heat-inactivated fetal bovine serum (FBS) (Invitrogen), 1% penicillin-streptomycin (Invitrogen) and 10 ng/mL doxycycline (Sigma, Stockholm, Sweden). For experiments, cells were grown for 24 to 168 hours prior to analysis in phenol red-free RPMI 1640 medium supplemented with 2% heat-inactivated FBS, 1% penicillin-streptomycin, 10 ng/mL -ERβ or 0.01 ng/mL doxycycline +ERβ in the presence of vehicle alone, ethanol and/or dimethyl sulfoxide (Sigma), or in 10 nM 2,3-bis(4-hydroxy-phenyl)-propionitrile (DPN) (Tocris, Bristol, United Kingdom), 10 nM 4,4',4"-(4-propyl-[1*H*]-pyrazole-1,3,5-triyl)*tris*phenol (PPT) (Tocris), 10 nM 17β-estradiol (E2) (Sigma), 10 nM 7-bromo-2-(4-hydroxyphenyl)-1,3-benzoxazol-5-ol (WAY) (Tocris), 100 nM ICI 182, 789 (ICI) (Tocris) or 100 to 1,000 nM 4-hydroxy-tamoxifen (4-OH-T) (Sigma). MCF-7 breast cancer cells with tetracycline-regulated expression of ERβ485 were treated in a similar manner. T47-DPBI cells (Mock) were used as controls.

### Quantitative real-time polymerase chain reaction assays

Cells were grown in six-well tissue culture plates for 24 to 96 hours and lysed in TRIzol reagent (Invitrogen), then RNA was extracted and cDNA was synthesized as described previously [46]. Quantitative real-time polymerase reaction assays (qRT-PCR) were performed with SYBR Green PCR Master Mix in an ABI PRISM 7500 (Applied Biosystems, Foster City, USA). The following primers were used: *18S *forward 5'-CCTGCGGCTTAATTTGACTCA-3', reverse 5'-AGCTATCAATCTGTCAATCCTGTCC-3'; *ERβ *forward 5'-ACTTGCTGAACGCCGTGACC-3', reverse 5'-CAGATGTTCCATGCCCTTGTT-3'; *HER2 *forward 5'-AAAGGCCCAAGACTCTCTCC-3', reverse 5'-CAAGTACTCGGGGTTCTCCA-3'; *HER3 *forward 5'-GTCATGAGGGCGAACGAC-3', reverse 5'-AGAGTCCCAGGACACACTGC-3'; and *PTEN *forward 5'-GGGGAAGTAAGGACCAGAGAC-3', reverse 5'-TCCAGATGATTCTTTAACAGGTAGC-3'. Quantification was carried out following the supplier's protocols using the standard curve method.

### Whole-cell extracts

Cells grown on plates were washed with ice-cold phosphate-buffered saline (PBS), transferred to Eppendorf tubes and pelleted by centrifugation. Cell pellets were freeze-thawed and resuspended with PBS-TDS buffer (PBS with 1% Triton X-100, 0.5% sodium deoxycholate, 0.1% sodium dodecyl sulfate (SDS), 1 mM ethylenediaminetetraacetic acid and phosphatase inhibitors (Sigma)), incubated for 30 minutes on ice and centrifuged at 11,000 rpm for 10 minutes at 4°C. Supernatants were collected for further analysis. Protein quantification was carried out using a bicinchoninic acid protein assay kit (Pierce Biotechnology, Rockford, USA).

### Western blot analysis

Forty micrograms of total cellular protein were separated using 7.5% SDS-polyacrylamide gel electrophoresis and electrotransferred onto a nitrocellulose membrane (Hybond-C; AmershamBuckinghamshire, United Kingdom). After blocking in 5% milk protein (wt/vol) in PBS, 0.1% Tween 20 (vol/vol) membranes were sequentially incubated with primary and secondary antibodies. The following antibodies were used: anti-ERβ (14021; Abcam, Cambridge, United Kingdom), GTX110607 (GeneTex, Irvine, USA), anti-phospho-HER3 tyr1289 (21D3; Cell Signaling Technology, Danvers, USA), anti-phospho-Akt pathway sampler kit (9916; Cell Signaling Technology), anti-phospho-HER2 antibody sampler kit (9923; Cell Signaling Technology), anti-PTEN (26H9; Cell Signaling Technology); anti-α-tubulin (11H10; Cell Signaling Technology), anti-EGFR (E12020; Transduction Laboratories, Franklin Lakes, USA), anti-HER3 (sc-285; Santa Cruz Biotechnology, Santa Cruz, CA, USA) and anti-β-actin (Sigma). The secondary antibodies were horseradish peroxidase-conjugated (Sigma). Visualization was carried out using the ECL Plus kit (Amersham) or the SuperSignal West Pico kit (Pierce Biotechnology). At least three independent experiments were carried out.

### Immunofluorescence

Cells were cultured on sterilized glass coverslips in high- or low-doxycycline conditions for 4 days as described above. The cells were fixed by ice-cold methanol and ice-cold acetone for 10 minutes and 1 minute, respectively. Blocking of nonspecific binding was done with BlockAce (Dainippon Pharmaceutical, Osaka, Japan) for 1 h at room temperature. The samples were then incubated overnight at 4°C with the following antibodies at the indicated dilutions in 10% BlockAce in PBS: anti-HER2, 1:150 (29D8; Cell Signaling Technology), and anti-PTEN, 1:100 (26H9; Cell Signaling Technology). After washes with PBS, samples were incubated with corresponding Alexa Fluor 568-conjugated secondary antibody 1:500 (Invitrogen) and Hoechst 33342 5 μg/mL (Molecular Probes, Eugen, USA) in PBS for 1 hour at room temperature. Samples were mounted with VECTASHIELD (Vector Laboratories, Burlingame, USA) after washes with PBS. Negative controls were incubated without primary antibody. To compare staining intensity between different samples, pictures were obtained with fixed exposure time. Staining was repeated three times to confirm consistent results.

### Fluorescence imaging

Pictures of fluorescence staining were captured with a Zeiss Axioplan 2 microscope using Zeiss Plan-Apochromat 63×/1.40 oil lens (Carl Zeiss, Oberkochen, Germany). Images were acquired with a Zeiss AxioCam MRm camera under the same settings. Captured images were processed using the AxioVision Rel 4.6 program and edited using Adobe PhotoShop C54 software (Adobe, San Jose, USA), and the same adjustments were applied to all images.

### Cell proliferation

T47-DERβ and MCF-7ERβ cells were cultured for 3 days in high (-ERβ) or low (+ERβ) doxycycline concentrations in the absence or presence of vehicle, E2 or WAY. On the third day, cells were replated on 96-well plates and allowed to adhere for 24 hours. Thereafter increasing concentrations of 4-OH-T were added. Growth medium was changed every other day. Cell viability was measured after 0, 5 and 7 days of incubation with 4-OH-T using a colorimetric assay (WST-1; Roche, Basel, Switzerland) following the manufacturer's suggestions. Measurement of absorbance was done using a SpectraMax 250 microplate reader (Molecular Devices, Sunnyvale, USA) against a background control as blank.

### Statistical analysis

Differences between more than two groups were compared by one-way analysis of variance and Tukey's multiple posttest using GraphPad software (GraphPad, San Diego, USA).

## Results and discussion

### AKT signaling is repressed by ERβ

**Figure 1:**
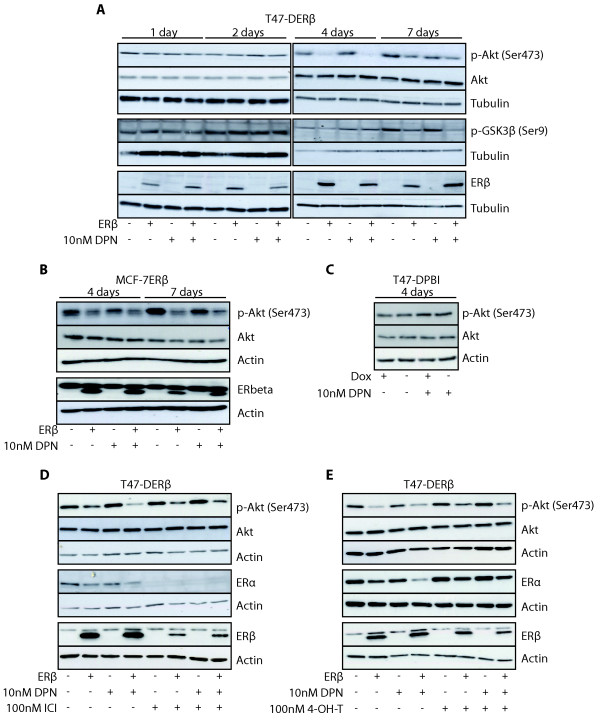
**Estrogen receptor β expression decreases Akt signaling in breast cancer cells**. **(A) **T47-DERβ cells were treated for 1 day up to 7 days with 10 ng/mL (-ERβ) or 0.01 ng/mL (+ERβ) doxycycline in the presence or absence of 10 nM 2,3-bis(4-hydroxy-phenyl)-propionitrile (DPN). **(B) **MCF-7ERβ cells were treated for 4 days or 7 days with 10 ng/mL (-ERβ) or 0.01 ng/mL (+ERβ) doxycycline in the presence or absence of 10 nM DPN. **(C) **T47-DPBI control cells were treated for 4 days with 10 ng/mL or 0.01 ng/mL doxycycline, in the presence or absence of 10 nM DPN. **(D and E) **T47-DERβ cells were treated for 4 days with 10 ng/mL (-ERβ) or 0.01 ng/mL (+ERβ) doxycycline in the presence or absence of **(D) **10 nM DPN with or without 100 nM ICI 182, 789 (ICI) or **(E) **100 nM 4-hydroxy-tamoxifen (4-OH-T). For Figures 1A to 1E, cell lysates were analyzed by immunoblotting. In Figure 1A, the estrogen receptor β (ERβ) antibody used was 14021 from Abcam. In Figures 1B, 1D and 1E, the ERβ antibody was GTX110607 from GeneTex. This antibody produced a nonspecific band above the ERβ band.

To assess the effect of ERβ on Akt signaling in human breast cancer cells, ERα-expressing T47-D and MCF-7 cells with inducible expression of ERβ (T47-DERβ and MCF-7ERβ, respectively) were grown at inducing conditions for different times, and active Akt as well as the activity of a downstream target were investigated by immunoblot analysis. Both cell lines used in the present study have *PIK3CA *mutations, H1047R (catalytic domain) in T47-D and E545K (helical domain) in MCF-7 cells [47], resulting in active Akt, higher in T47-D, at low stimulatory conditions. In both cell lines, expression of ERβ clearly downregulated phosphorylated Akt (pAkt) (Additional file 1). To further analyze the ERβ effect, pAkt levels were assessed throughout 1 to 7 days (Figure 1A). In T47-DERβ cells, levels of pAkt were clearly downregulated by ERβ after 4 and 7 days of ERβ induction (Figure 1A). No additional effect was seen upon the addition of the selective ERβ agonist DPN. Levels of total Akt protein did not change, indicating that reduced pAkt levels were due to less phosphorylation. Downregulation of pAkt was also observed upon ERβ expression in MCF-7ERβ cells (Figure 1B), showing that this is not a unique ERβ effect in one selected T47-D cell clone. Moreover, pAkt levels in the mock cell line T47-DPBI were not affected by different doxycycline concentrations (Figure 1C), indicating that levels of pAkt are influenced not by doxycycline, but by induction of ERβ expression. One downstream target of Akt is GSK3β. Following ERβ expression, pAkt downregulation correlated with reduced levels of phosphorylated GSK3β (Figure 1A).

Since addition of the ERβ ligand DPN exerted no stable, repeatable additional effect to that already observed following ERβ expression (Figure 1 and 1 data not shown), we investigated whether ER antagonists would prevent ERβ-induced decrease of Akt phosphorylation. For this purpose, ICI 182, 780 (ICI), a selective ER downregulator, and the selective estrogen modulator 4-OH-T were used. As expected, ICI induced complete downregulation of ERα (Figure 1D). ERβ protein levels were partially downregulated by ICI, whereas 4-OH-T had no significant effect on either ERα or ERβ protein levels (Figure 1E). Furthermore, ERα protein levels were reduced in cells expressing ERβ (Figures 1D and 1E). This latter finding was consistently observed in all inducible systems that we tested. Treatment with ICI or 4-OH-T did not inhibit the ERβ-induced decrease of pAkt levels. However, in ICI- or 4-OH-T-treated cells, the ERβ-induced decrease of pAkt levels was less than that in cells not exposed to ICI or 4-OH-T, suggesting a weak antagonistic action of ICI and 4-OH-T. In summary, in two different ERα-expressing human breast cancer cell lines, ERβ expression clearly reduced activation of the Akt signaling pathway.

### ERβ regulation of HER2 and HER3 expression

**Figure 2:**
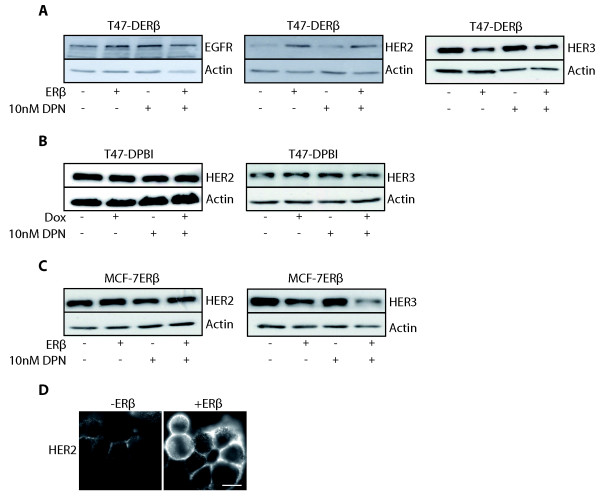
**Effect of ERβ on expression of receptor tyrosine kinases upstream of Akt**. **(A) **T47-DERβ cells. EGFR, epidermal growth factor receptor; HER2, proto-oncogene *c-ErbB-2*; HER3, receptor tyrosine kinase erbB-3. **(B) **T47-DPBI cells **(C) **MCF-7ERβ cells. For Figures 1A to 1C, cells were treated for 4 days with 10 ng/mL or 0.01 ng/mL doxycycline in the presence or absence of 10 nM DPN and cell lysates were analyzed by immunoblotting. **(D) **Immunofluorescence study of T47-DERβ cells treated for 4 days with 10 ng/mL (-ERβ) or 0.01 ng/mL (+ERβ) doxycycline. Cells were stained with anti-HER2 antibody. Scale bar, 10 μm.

Members of the EGFR family are potent activators of the PI3K/Akt pathway, contributing to endocrine resistance in breast tumors. Therefore, we investigated the effect of ERβ expression on EGFR, HER2 and HER3 protein levels in T47-D and MCF-7 cells. Upon ERβ expression, levels of EGFR were unchanged in T47-DERβ cells (Figure 2A). In contrast, ERβ expression upregulated HER2 and downregulated HER3 protein levels. No further changes were seen with DPN. Treatment of T47-DERβ cells expressing ERβ or not with the ERα-selective ligand PPT decreased HER2 protein expression but had no effect on HER3 protein expression (Additional file 2). In the control mock cell line T47-DPBI, the two doxycycline concentrations did not change HER2 and HER3 protein levels (Figure 2B), suggesting that expression of these two membrane receptors in T47-DERβ cells is regulated by ERβ and not by doxycycline. In MCF-7ERβ cells, expression of HER2 protein was not clearly upregulated by ERβ. However, HER3 protein expression was downregulated in MCF-7ERβ upon ERβ expression (Figure 2C), showing that ERβ-induced HER3 downregulation is not seen in only one isolated cell clone and type. The ERβ-induced increase in HER2 levels was also seen by performing immunocytochemistry in T47-DERβ cells (Figure 2D).

**Figure 3:**
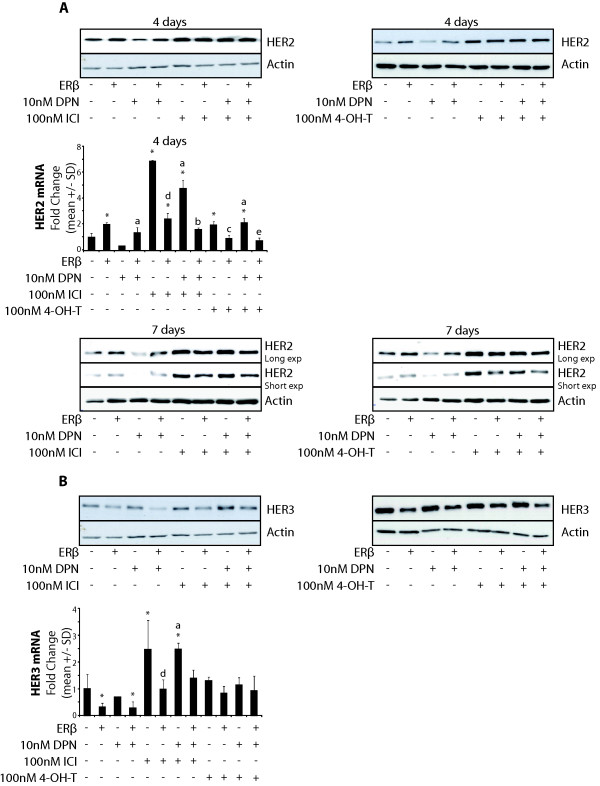
**Effect of ERβ on HER2 and HER3 protein and mRNA expression**. **(A) **T47-DERβ cells were treated for 4 or 7 days with 10 ng/mL (-ERβ) or 0.01 ng/mL (+ERβ) doxycycline in the presence or absence of 10 nM DPN with or without 100 nM ICI or 100 nM (4-OH-T), and lysates were analyzed by immunoblotting. Also, cells were analyzed for HER2 mRNA expression by performing quantitative polymerase chain reactions (qPCRs). **(B) **T47-DERβ cells were treated for 4 days with 10 ng/mL (-ERβ) or 0.01 ng/mL (+ERβ) doxycycline in the presence or absence of 10 nM DPN with or without 100 nM ICI or 100 nM 4-OH-T, and lysates were analyzed by immunoblotting. Also, cells were analyzed for HER3 mRNA expression by performing qPCR. Significant differences were analyzed using one-way analysis of variance (ANOVA) and Tukey's multiple posttest: *P < 0.05 vs. -ERβ, ^a^*P *< 0.05 vs. -ERβ + DPN, ^b^*P *< 0.05 vs. -ERβ + DPN + ICI, ^c^*P *< 0.05 vs. -ERβ + 4-OH-T, ^d^*P *< 0.05 vs. -ERβ + ICI, ^e^*P *< 0.05 vs. -ERβ + DPN + 4-OH-T.

Exposure of cells to ICI or 4-OH-T induced an overall increase in expression of HER2 protein, and ERβ upregulation of HER2 was completely abolished by both ICI and 4-OH-T, as analyzed after 4 days of ERβ expression (Figure 3A). qPCR analysis showed that in cells not exposed to ICI or 4-OH-T, ERβ expression increased HER2 mRNA levels. Upon ICI or 4-OH-T exposure, HER2 mRNA levels increased, and, in this experimental setting, ERβ decreased HER2 mRNA levels. This was not clearly related to HER2 protein levels at 4 days of ERβ expression. However, following 7 days of ERβ expression, HER2 protein levels correlated with HER2 mRNA levels seen at 4 days, indicative of slow turnover of HER2 protein (Figure 3A, bottom). In a recent study, PAX2 and the ER coactivator AIB1 (SRC-3) were shown to compete for binding to ERα and regulation of *HER2 *gene transcription in breast cancer cells [48]. In cells treated with estrogen or tamoxifen, PAX2 acted together with ERα as a transcriptional repressor where HER2 mRNA was decreased, whereas HER2 transcription increased with high levels of SRC-3. Interestingly, we show here that HER2 mRNA and protein levels were increased upon ERβ expression. E2 treatment or treatment with the ERα-selective ligand PPT of T47-DERβ cells with no ERβ induction resulted in downregulation of HER2 protein levels (Additional files 2 and 3), indicating that under our experimental conditions, ERα exerted a repressive effect. Thus, we hypothesize that ERβ upregulation of HER2 could be related to reduced ERα action by the formation of ERα/ERβ heterodimers, which relieves ERα homodimer-mediated repression of HER2 expression, and/or by ERβ repressing the expression of ERα, as seen in our cell models. Exposure of T47-DERβ cells to the ER antagonists ICI or 4-OH-T enhanced HER2 mRNA and protein levels, an effect that could be due to removal of the ERα-PAX2 repressive effect on *HER2 *gene expression. Interestingly, when ICI or 4-OH-T was added to ERβ expressing cells, ERβ changed from being an inducer to a repressor of HER2 mRNA and protein expression. In our cell model, exposure to ICI resulted in the disappearance of ERα protein and a marked increase of HER2. Thus, in ICI-treated cells expressing ERβ, it is likely that an ERβ homodimer is acting as a repressor of *HER2 *gene expression. Furthermore, 4-OH-T treatment also shifted ERβ from being an activator to being a repressor. This could perhaps be explained by 4-OH-T having an antagonistic action on ERα but not on ERβ, a possible scenario if both receptors are activated ligand-independently by phosphorylation, where ERβ is less sensitive to antagonism from 4-OH-T [49]. It is clear that further studies with T47-DERβ cells are needed to better understand the mechanisms by which ERβ regulates *HER2 *expression, including determination of PAX2 and SRC-3 levels under different experimental conditions.

Neither ICI nor 4-OH-T prevented ERβ-induced downregulation of HER3 protein levels (Figure 3B). qRT-PCR analysis showed that ICI and 4-OH-T both increased overall HER3 mRNA levels, which could be indicative of ERα, similarly to ERβ, having a repressive effect on HER3 mRNA expression. However, the ERα-selective ligand PPT had no effect on HER3 protein expression. Further studies are needed to explain this difference. ICI, but not 4-OH-T, clearly did not inhibit ERβ-induced downregulation of HER3 mRNA. The ICI-induced increase and ERβ-induced downregulation of HER3 mRNA levels in ICI-treated cells correlated well with HER3 protein levels. This was not obvious in 4-OH-T treated cells, where a difference was seen at the protein level but not at the mRNA level.

### ERβ downregulates heregulin-induced activation of HER2/HER3 dimer and Akt

Heregulin-β1 (HRG-β1), a member of the EGFR family, is a ligand for HER3. As HER3 has no intracellular tyrosine kinase domain, it partners with other members of the EGFR family to initiate intracellular signaling. The preferred dimerization partner is HER2, which has tyrosine kinase activity. In the intracellular domain of HER3, there are six tyrosines that, upon phosphorylation by HER2, will serve as docking sites for the p85 adaptor subunit of PI3K. Thus, HRG-β1 activation of the HER2/HER3 dimer results in strong activation of the PI3K/Akt signaling pathway.

**Figure 4:**
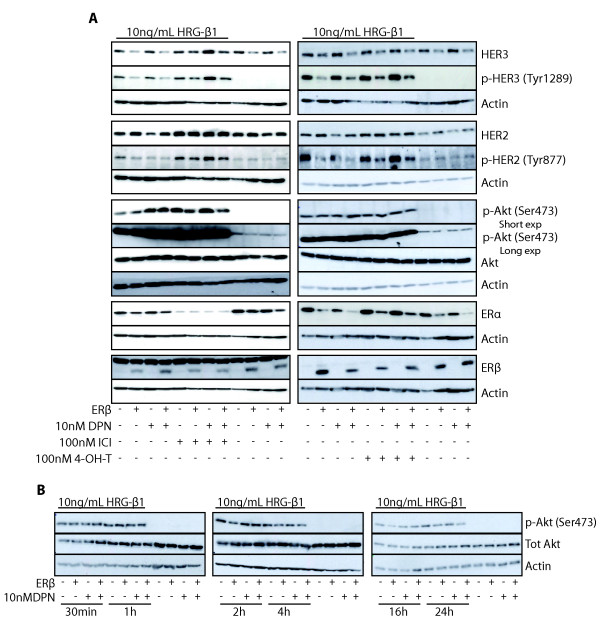
**Effect of ERβ on heregulin-β1-activated HER2, HER3 and Akt**. **(A) **T47-DERβ cells were treated for 4 days with 10 ng/mL (-ERβ) or 0.01 ng/mL (+ERβ) doxycycline in the presence or absence of 10 nM DPN with or without 100 nM ICI or 100 nM 4-OH-T and stimulated with heregulin-β1 (HRG-β1) for 30 minutes. **(B) **T47-DERβ cells were treated as described above and stimulated with HRG-β1 for 30 minutes up to 24 hours. Lysates were analyzed by immunoblotting.

To elucidate whether ERβ could influence HRG-β1 activation of the HER2/HER3 and Akt pathways, T47-DERβ cells were cultured for 4 days with or without ERβ expression and in the absence or presence of DPN, whereafter HRG-β1 was added for 30 minutes. The addition of HRG-β1 to T47-DERβ cells clearly induced phosphorylation of HER2, HER3 and Akt (Figure 4A). ERβ decreased levels of phosphorylated HER3 (Figure 4A). This effect probably could be explained by the ERβ-induced downregulation of HER3 protein (Figures 2A, D, 3B and 4A). Interestingly, even though ERβ upregulated HER2 protein levels (Figures 2A, C, 3A and 4A), ERβ decreased HRG-β1-induced HER2 phosphorylation, also possibly due to ERβ-induced decrease of the HER2 dimerization partner HER3.

Exposure of T47-DERβ cells to HRG-β1 for 30 minutes also dramatically increased levels of pAkt (Figure 4A). At this time point, ERβ expression did not decrease levels of phosphorylated Akt. However, a time study of HRG-β1-stimulated cells showed that from 2 hours onward, ERβ presence decreased levels of phosphorylated Akt (Figure 4B). One possible explanation for this could be that in the acute phase after HRG-β1 addition, there was a massive activation of Akt due to the already mutated *PIK3CA *in T47-DERβ cells, an activation that ERβ could not inhibit. However, ERβ could decrease levels of phosphorylated Akt after its peak activity, when the activity was still clearly above that in unstimulated cells (Figure 4A).

Exposure of cells to DPN, E2 or WAY (another ERβ selective agonist) did not influence levels of HRG-β1-induced phosphorylated HER2, HER3 and Akt (Figure 4A andAdditional file 3). To further investigate the Akt pathway in the context of endocrine sensitivity and ERβ expression, in addition to HRG-β1 treatment, cells were further treated with ICI or 4-OH-T (Figure 4A). ICI and 4-OH-T exposure both increased levels of phosphorylated HER2 and HER3 in the absence or presence of ERβ. An effect that may be related to increased total HER2 levels in cells treated with ICI or 4-OH-T (Figures 3A and 4A). However, levels of phosphorylated HER2 and pHER3 were clearly lower when ERβ was present.

### PTEN levels increase following ERβ expression

**Figure 5:**
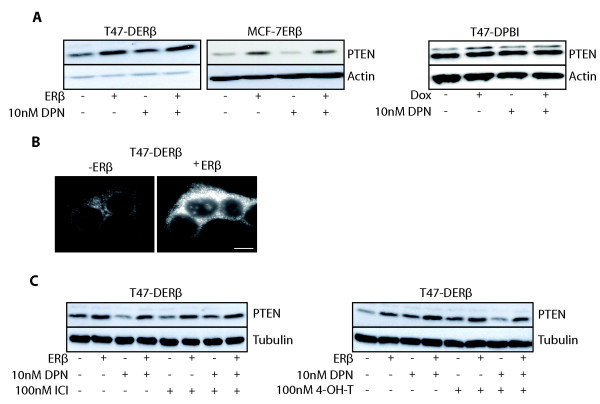
**PTEN levels increase upon ERβ expression**. (A) T47-DERβ cells, MCF-7ERβ cells and T47-DPBI control cells were treated for 4 days with 10 ng/mL or 0.01 ng/mL doxycycline in the presence or absence of 10 nM DPN, and lysates were analyzed by immunoblotting. **(B) **Phosphatase and tensin homologue deleted on chromosome 10 (PTEN) immunofluorescence of T47-DERβ cells treated for 4 days with 10 ng/mL (-ERβ) or 0.01 ng/mL (+ERβ) doxycycline. Scale bar, 10 μm. **(C) **T47-DERβ cells were treated for 4 days with 10 ng/mL (-ERβ) or 0.01 ng/mL (+ERβ) doxycycline in the presence or absence of 10 nM DPN with or without 100 nM ICI or 100 nM 4-OH-T, and lysates were analyzed by immunoblotting.

PTEN mediates its main tumor-suppressive function through dephosphorylation of PIP3. Interestingly, in a recent report [50], it was shown that in mice with a subtle reduction of PTEN expression (80% of the normal level), different types of tumors developed with mammary carcinomas occurring at highest penetrance. Also, reduced levels or loss of PTEN has been implicated in the development of endocrine resistance in breast cancer. Since PTEN is an important regulator of Akt signaling, we found it important to investigate the effect of ERβ on PTEN expression. ERβ expression upregulated PTEN levels in both T47-DERβ and MCF-7ERβ cells (Figure 5A). DPN, E2 or WAY addition did not further upregulate PTEN levels (Figure 5A and Additional file 4). No significant changes were seen in the control cell line (T47-DPBI) under the same conditions (Figure 5A). Thus, the ERβ effect on PTEN levels was seen not only in one breast cancer cell type and was not due to doxycycline. The T47-DERβ cells expressing ERβ or not were also analyzed using PTEN immunofluorescence. As shown in Figure 5B, PTEN protein levels were clearly upregulated in ERβ-expressing cells. Exposure of T47-DERβ cells to ICI or 4-OH-T did not decrease or inhibit the ERβ effect on PTEN protein levels (Figure 5C and Additional file 5). The effect of ERβ expression on PTEN mRNA levels was also investigated. However, no conclusive data could be obtained from these experiments (data not shown). One explanation for the observed effects of ERβ on PTEN protein levels could be that ERβ regulates expression of other proteins that in turn regulate PTEN. Further studies are needed to clarify this hypothesis.

### Expression of ERβ sensitizes breast cancer cells to tamoxifen

**Figure 6:**
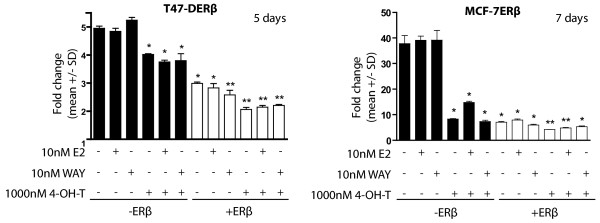
**ERβ improves sensitivity to tamoxifen in breast cancer cells**. T47-DERβ cells and MCF-7ERβ cells were treated for 5 and 7 days, respectively, with 10 ng/mL (-ERβ) or 0.01 ng/mL (+ERβ) doxycycline in the presence or absence of 10 nM 7-bromo-2-(4-hydroxyphenyl)-1,3-benzoxazol-5-ol (WAY) or 10 nM 17β-estradiol (E2). Thereafter cells were exposed to 1,000 nM 4-OH-T. At indicated time points, cell numbers were measured using a WST-1 colorimetric assay as described in Materials and methods. Significant differences were analyzed using one-way ANOVA and Tukey's multiple posttest. *P < 0.05 vs. -ERβ. **P < 0.05 vs. +ERβ.

PTEN downregulation as well as increased HER2/HER3 and Akt signaling have been associated with endocrine resistance in breast tumors. With our above-described results in mind, we found it imperative to investigate whether expression of ERβ would increase the sensitivity to tamoxifen in T47-DERβ and MCF-7ERβ breast cancer cells. Experiments were performed in cells where ERβ was expressed for 4 days in the absence or presence of the agonists E2 and WAY, whereafter 1,000 nM tamoxifen was added for either 5 days (T47-DERβ) or 7 days (MCF-7ERβ). The selective agonist DPN was not used in these experiments because of our previous findings that, besides inhibiting proliferation, DPN also seems to affect this kind of viability assay, depending on metabolism [51]. Instead, the selective ERβ ligand WAY was used, which did not influence the assay. In both cell lines, and in the absence of ERβ, 4-OH-T decreased growth (Figure 6). In MCF-7ERβ cells, we observed a more marked effect, which could be due to less active Akt signaling (Additional file 1). In MCF-7ERβ cells, but not in T47-DERβ cells, E2 also slightly counteracted the effect of 4-OH-T. Expression of ERβ alone clearly reduced growth in both cell lines. This was further significantly enhanced with exposure to WAY in ERβ-expressing T47-DERβ cells. In MCF-7ERβ cells, a slight enhancement of growth reduction was also seen with WAY treatment, but it did not reach significance. Expression of ERβ, together with exposure to 4-OH-T, significantly further decreased cell growth as compared to growth seen in only ERβ-expressing cells. Similar results were seen in both cell lines with 500 nM tamoxifen (results not shown). In summary, these results show that ERβ expression render ERα-expressing breast cancer cells more sensitive to tamoxifen treatment.

In several experiments, the addition of agonist or antagonist did not increase or decrease the effect of ERβ expression on its own. This could indicate that in these breast tumor cells, ERβ is activated in a ligand-independent manner; for example, it is phosphorylated in the AF-1 domain and then is also less inhibited by antagonists that have a focus on ligand-binding and the AF-2 domain [49]. Further studies are needed to clarify this hypothesis with mapping of phosphorylated sites of ERβ in these cells. Interestingly, a recent report [52] shows that ERβ phosphorylated at serine 105 is associated with a good prognosis in breast cancer. A future challenge is to develop ligands that, in this setting, that is, ERβ-expressing breast cancers with increased kinase activity, could activate or increase the inhibitory effect of ERβ on Akt signaling.

## Conclusions

Our results suggest a link between expression of ERβ and endocrine sensitivity by increasing PTEN levels and decreasing HER2/HER3 signaling, thereby reducing Akt signaling with subsequent effects on proliferation, survival and tamoxifen sensitivity of breast cancer cells. This study supports initiatives to further investigate whether ERβ presence in breast cancer samples is an indicator for endocrine response. Current therapies in ERα-positive breast cancers aim to impair ERα activity with antagonists or by removal of endogenous estrogens with aromatase inhibitors. Data from this study could be taken as indicative for using ERβ as a target in selected groups of breast cancer.

## Abbreviations

DPN: 2,3-bis(4-hydroxy-phenyl)-propionitrile; EGFR: epidermal growth factor receptor; ER: estrogen receptor; E2: 17β-estradiol; HER2 proto-oncogene c-ErbB-2; HER3: receptor tyrosine kinase erbB-3; HRG-β1: heregulin-β1; ICI: ICI 182: 789; 4-OH-T: 4-hydroxy-tamoxifen; PCR: polymerase chain reaction; PI3K: phosphatidylinositol 3-kinase; PIP3: phosphatidylinositol (3,4,5)-triphosphate; PPT: 4,4',4"-(4-propyl-[1*H*]-pyrazole-1,3,5-triyl)*tris*phenol; PTEN: phosphatase and tensin homologue deleted on chromosome 10; qPCR: quantitative real-time polymerase chain reaction; RTK: receptor tyrosine kinase; WAY: 7-bromo-2-(4-hydroxyphenyl)-1,3-benzoxazol-5-ol.

## Competing interests

JÅG received a consultancy honorarium and has an ownership interest in KaroBio AB and is a consultant for Bionovo. The other authors declare that they have no competing interests.

## Authors' contributions

KL participated in the study design and coordination; carried out the cell cultures, Western blot analysis and RT-PCR analyses; and drafted the manuscript. LH conceived of the study, participated in its design and helped to draft the manuscript. YO optimized, performed, analyzed and interpreted data from immunostaining and helped to draft the manuscript. JÅG participated in general discussions of the project, helped to draft the manuscript and revised it critically for important intellectual content. LAH conceived of the study, participated in the design of the study, performed some Western blot analyses and helped to draft the manuscript. All authors read and approved the final manuscript.

## Supplementary Material

Additional file 1 **Western blot analysis of Akt signaling in T47D and MCF-7 cells**. T47-DERβ and MCF-7ERβ cells were treated for 4 days with 10 ng/mL (-ERβ) or 0.01 ng/mL (+ERβ) doxycycline in the presence or absence of 10 nM 2,3-bis(4-hydroxy-phenyl)-propionitrile (DPN). Lysates were analyzed by immunoblotting. ERβ, estrogen receptor β. Click here for file

Additional file 2 **Effects of the ERα-selective ligand PPT on expression of HER2 and HER3**. T47-DERβ cells were treated for 4 days with 10 ng/mL (-ERβ) or 0.01 ng/mL (+ERβ) doxycycline, in the presence or absence of 10 nM 4,4¶,400-(4-propyl-[1*H*]-pyrazole-1,3,5-triyl)trisphenol (PPT). Lysates were analyzed by immunoblotting. Click here for file

Additional file 3 **Influence of different ER ligands on ERβ effects on HRG-β1-stimulated signaling**. T47-DERβ cells were treated for 4 days with 10 ng/mL (-ERβ) or 0.01 ng/mL (+ERβ) doxycycline in the presence or absence of 10 nM DPN, 10 nM 7-bromo-2-(4-hydroxyphenyl)-1,3-benzoxazol-5-ol (WAY) or 10 nM 17β-estradiol (E2) and thereafter were stimulated for 30 minutes with 10 ng/mL heregulin-β1 (HRG-β1). Lysates were analyzed by immunoblotting. Click here for file

Additional file 4 **ERβ effects on PTEN protein expression using different ER selective ligands**. T47-DERβ cells were treated for 4 days with 10 ng/mL (-ERβ) or 0.01 ng/mL (+ERβ) doxycycline in the presence or absence of 10 nM DPN, 10 nM WAY or 10 nM E2. Lysates were analyzed by immunoblotting. PTEN, phosphatase and tensin homologue deleted on chromosome 10. Click here for file

Additional file 5 **Influence of ER antagonists on ERβ effects on PTEN protein expression**. T47-DERβ cells were treated for 4 days with 10 ng/mL (-ERβ) or 0.01 ng/mL (+ERβ) doxycycline in the presence or absence of 10 nM DPN, 100 nM 4-OH-T or 100 nM ICI 182, 789 (ICI). Lysates were analyzed by immunoblotting. Densitometric scanning of three immunoblots is shown. Click here for file
