# Supplementary figures and images for: Estrogen receptor β represses Akt signaling in breast cancer cells via downregulation of HER2/HER3 and upregulation of PTEN: implications for tamoxifen sensitivity
Source: Breast Cancer Res. 2011 Apr 14;13(2):R43. doi: 10.1186/bcr2865 (PMC3219206; doi:10.1186/bcr2865)

S.1

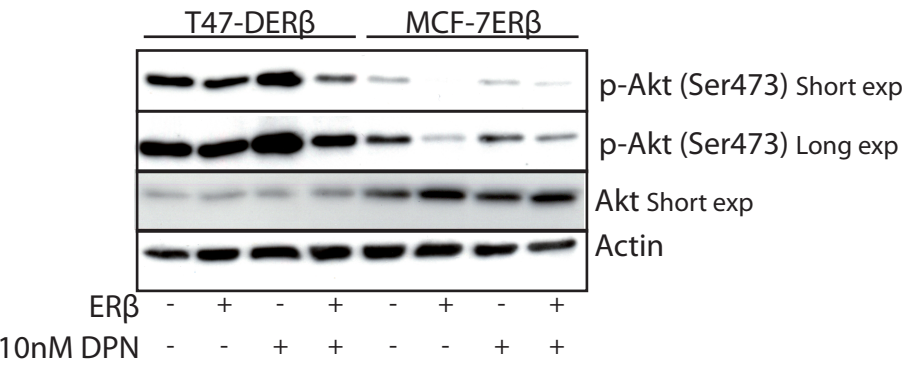

Supplement: Additional file 1 — Western blot analysis of Akt signaling in T47D and MCF-7 cells. T47-DERβ and MCF-7ERβ cells were treated for 4 days with 10 ng/mL (-ERβ) or 0.01 ng/mL (+ERβ) doxycycline in the presence or absence of 10 nM 2,3-bis(4-hydroxy-phenyl)-propionitrile (DPN). Lysates were analyzed by immunoblotting. ERβ, estrogen receptor β. [file bcr2865-S1.PDF]

S.2

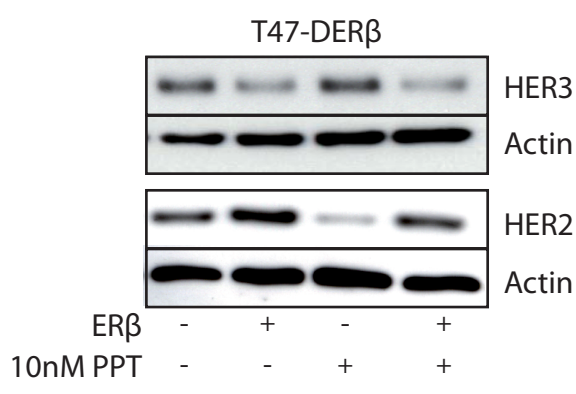

Supplement: Additional file 2 — Effects of the ERα-selective ligand PPT on expression of HER2 and HER3. T47-DERβ cells were treated for 4 days with 10 ng/mL (-ERβ) or 0.01 ng/mL (+ERβ) doxycycline, in the presence or absence of 10 nM 4,4¶,400-(4-propyl-[1H]-pyrazole-1,3,5-triyl)trisphenol (PPT). Lysates were analyzed by immunoblotting. [file bcr2865-S2.PDF]

S.3

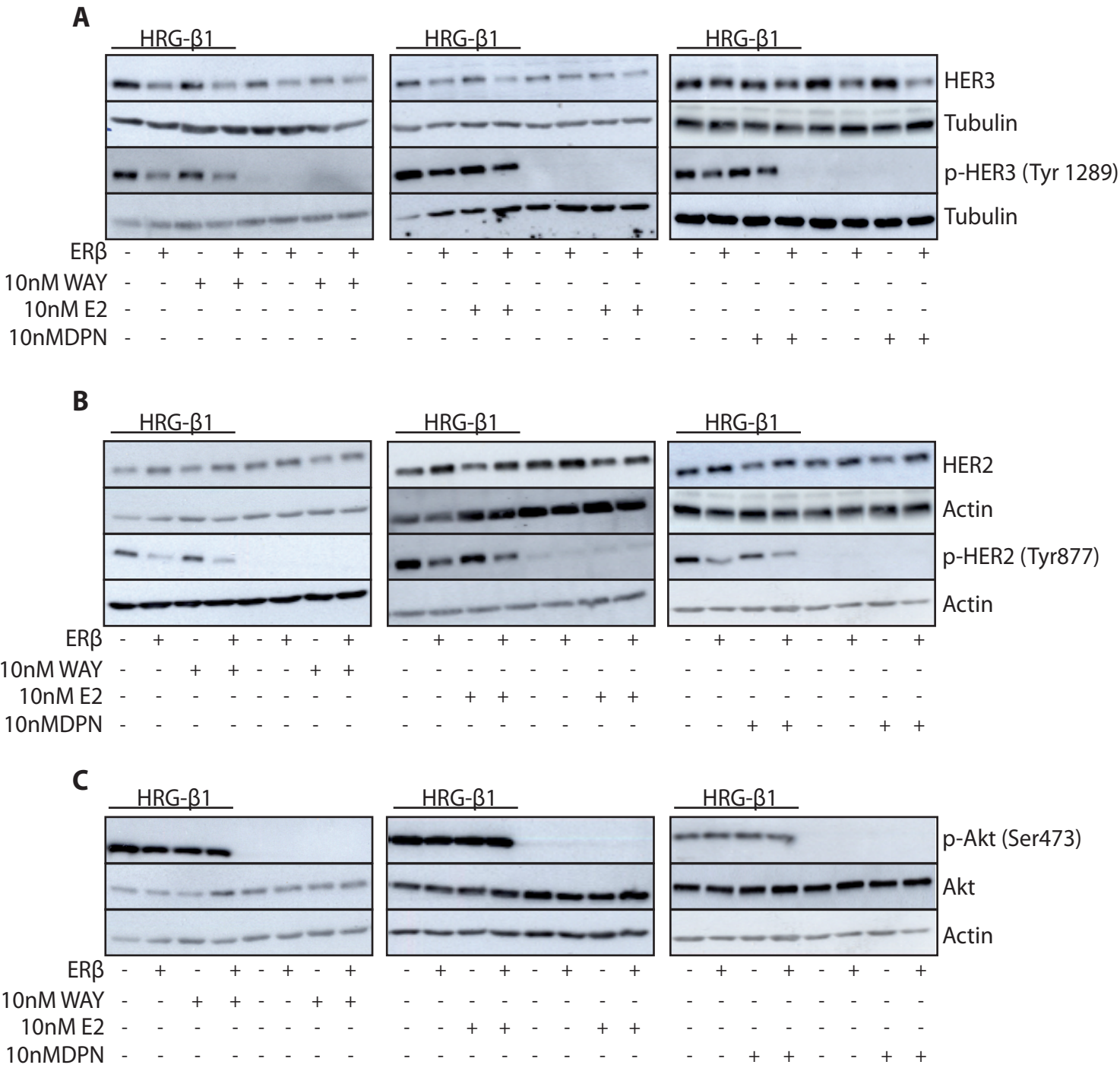

Supplement: Additional file 3 — Influence of different ER ligands on ERβ effects on HRG-β1-stimulated signaling. T47-DERβ cells were treated for 4 days with 10 ng/mL (-ERβ) or 0.01 ng/mL (+ERβ) doxycycline in the presence or absence of 10 nM DPN, 10 nM 7-bromo-2-(4-hydroxyphenyl)-1,3-benzoxazol-5-ol (WAY) or 10 nM 17β-estradiol (E2) and thereafter were stimulated for 30 minutes with 10 ng/mL heregulin-β1 (HRG-β1). Lysates were analyzed by immunoblotting. [file bcr2865-S3.PDF]

S.4

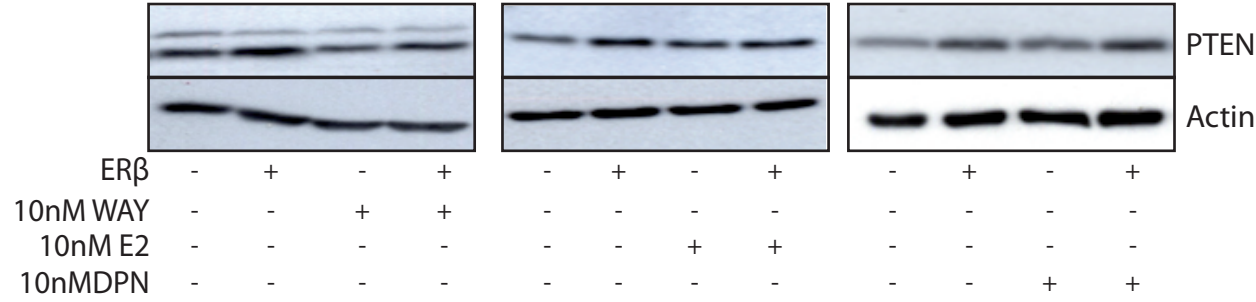

Supplement: Additional file 4 — ERβ effects on PTEN protein expression using different ER selective ligands. T47-DERβ cells were treated for 4 days with 10 ng/mL (-ERβ) or 0.01 ng/mL (+ERβ) doxycycline in the presence or absence of 10 nM DPN, 10 nM WAY or 10 nM E2. Lysates were analyzed by immunoblotting. PTEN, phosphatase and tensin homologue deleted on chromosome 10. [file bcr2865-S4.PDF]

S. 5

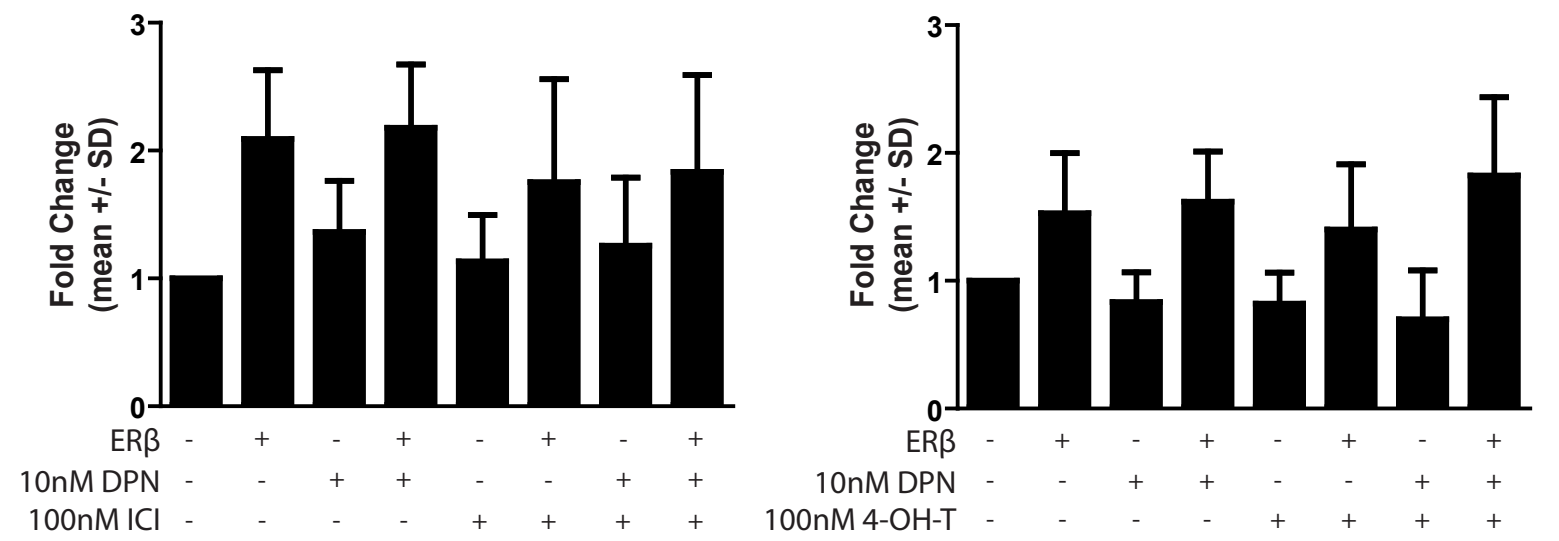

Supplement: Additional file 5 — Influence of ER antagonists on ERβ effects on PTEN protein expression. T47-DERβ cells were treated for 4 days with 10 ng/mL (-ERβ) or 0.01 ng/mL (+ERβ) doxycycline in the presence or absence of 10 nM DPN, 100 nM 4-OH-T or 100 nM ICI 182, 789 (ICI). Lysates were analyzed by immunoblotting. Densitometric scanning of three immunoblots is shown. [file bcr2865-S5.PDF]
